# Giant Cell Tumor of Soft Tissue—A Rare Cause of Mass in the Liver: A Case Report

**DOI:** 10.3389/fsurg.2022.830852

**Published:** 2022-04-27

**Authors:** Piaopiao Chen, Qiang Hu, Jinfeng Wu

**Affiliations:** Department of General Surgery, Tongde Hospital of Zhejiang Province, Hangzhou, China

**Keywords:** giant cell tumor of soft tissue, case report, primary liver tumor, rupture, hemorrhage

## Abstract

Primary giant cell tumors of soft tissues (GCT-STs) are extremely rare soft tissue tumors located both in superficial and in deep soft tissues. Clinically, GCT-ST manifests as a slow-growing, well-defined, painless mass. We report a case of an 88-year-old female patient with upper abdominal distension, fever, and anemia. Laparoscopic exploration revealed a tumor located in the left lobe of the liver with localized rupture and hemorrhage. Postoperative pathology revealed that the tumor was composed of monocytes and osteoclast-like multinucleated giant cells, accompanied by extensive hemorrhage, necrosis, and cytologic atypia. Because mitotic cells are difficult to be detected in pathological diagnosis, combined with immunohistochemistry, the tumor was diagnosed as a giant cell tumor of soft tissue. This case report highlights the primary choice of histology and immunohistochemistry for the correct diagnosis of GCT-ST because preoperative radiological diagnosis is nonspecific and prone to mistakes.

## Introduction

Giant cell tumor of soft tissue (GCT-ST) was first described in 1972 by Salm and Sissons (1). The majority of the GCT-ST cases occur in the extremities, trunk, head and neck, tendon sheaths, skeletal muscle, and skin (2), with the thigh being the most common site. Some tumors also occur in extraskeletal tissues, including the mediastinum, pancreas, liver, and thyroid. No primary giant cell tumor has been documented in the English literature; only the co-existence of osteoclast-like giant cells as an extremely rare variant of hepatocellular carcinoma has been reported in a few articles (3). Herein a case of hepatic giant cell tumor in an 88-year-old female, not associated with other hepatic tumors, is reported.

## Case Study

An 88-year-old female patient was admitted with a 3-day history of epigastric distension and low fever after exercise. The patient had no history of hepatitis and denied drinking and smoking, nor any family history attributable to liver disease, and no history of malignancy.

**Figure 1:**
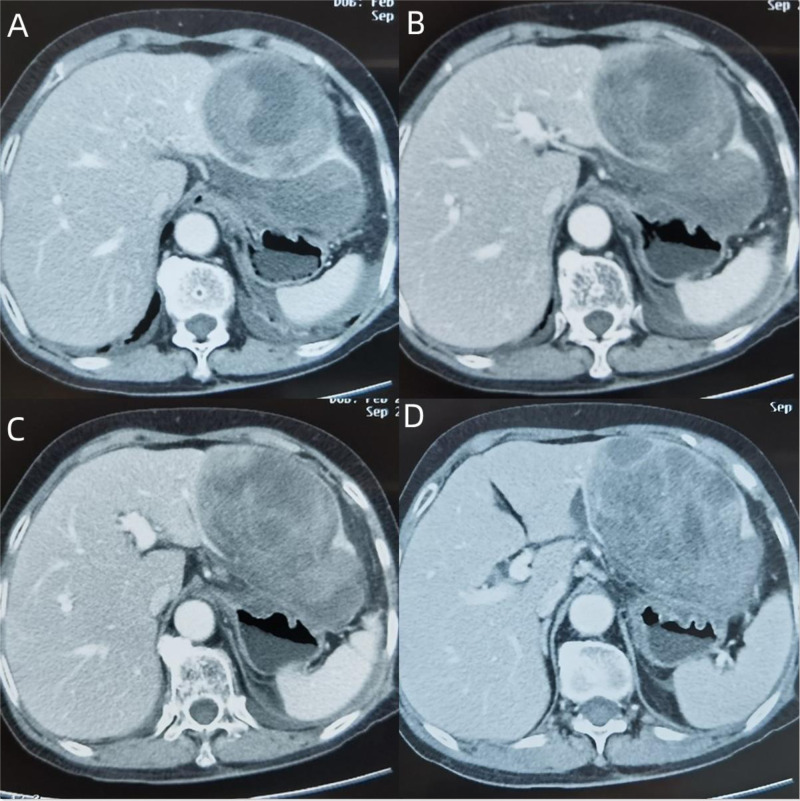
(**A**) The lesion is located in the left lateral lobe of the liver, mainly in III segment, part of II segment, with a clear realm, a heterogeneous focal mass of expansive growth, irregular patchy low-density areas of internal tissue, uneven strengthening of peripheral tissue, but the density is still lower than that of normal liver tissue. (**B**,**C**) The continuity of the lower edge of the tumor is interrupted, and there is a fluid accumulation in the hepatogastric space, which compresses the anterior wall of the stomach. (**D**) No enlarged lymph nodes are seen in the hepatic hilar, irregular cystic low-density areas appear at the inner edge of the tumor, and postoperative dissection confirms that the bile ducts are locally dilated.

Upon physical examination, the temperature was 37.7 ° and a large fixed nontender mass under the xiphoid process, mild tenderness in the upper abdomen, and no rebound pain and muscle tension were observed. The following results were obtained after the laboratory test: CBC: white blood cell 11.01×10^9^/L; hemoglobin 86 g/L; C-reactive protein 198.2 mg/L, and serum alpha-fetoprotein, carcinoembryonic antigen, and cancer antigens (CA19-9) were normal; liver function: total bilirubin, alanine transaminase, aspartate transaminase, alkaline phosphatase, and gamma-glutamyltransferase were normal, ALb 31.8 g/L; AMY was normal; fecal occult blood test was normal; and arterial blood gas analysis was clean. Enhanced CT of the upper abdomen showed left epigastric mass (Figure 1), measuring 12 cm × 10 cm, which probably occurred in the stomach and was considered a gastric stromal tumor. Gastroscopy showed that the gastric mucosa was elevated.

**Figure 2:**
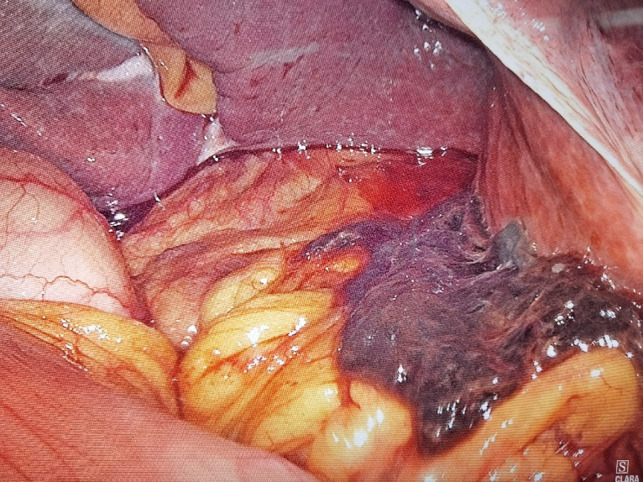
Massive blood clot located in the hepatogastric space surrounded by dark red unclotted blood.

**Figure 3:**
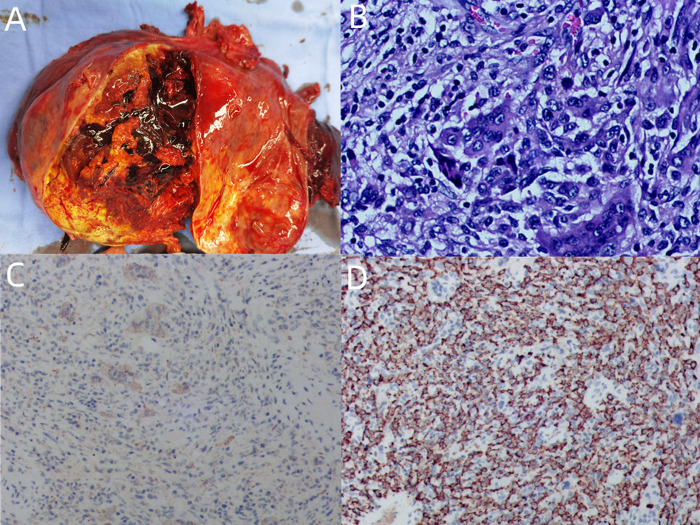
(**A**) General specimen: The mass is nodular, appears to have a fibrous envelope, has a clear perimeter, and is about 10 × 10 cm in size. (**B**) Tumor tissue (hematoxylin–eosin staining ×200): Tumor cells under the microscope are seen to consist of a mixture of monocyte-like cells and a large number of osteoclast-like multinucleated giant cells, with mild dysplasia of the nucleus, no pathological nuclear division, accompanied by extensive bleeding and necrosis. (**C**) GCT-STs are positive for immunostain CD68 (immunoperoxidase ×100). (**D**) GCT-STs are positive for immunostain CD163 (immunoperoxidase ×100).

Given the patient’s upper abdominal pain after exercise, accompanied by fever, decreased Hb, and markedly increased white blood cell and C-reactive protein, the mass was thought to be caused by local rupture and hemorrhage, combined with infection of hydrops abdominis. Provided the rupture and hemorrhage of the mass, laparoscopic exploration was performed. It was confirmed intraoperatively that the mass was located in the left lobe of the liver. There existed numerous blood clots in the liver and stomach space, surrounded by dark red unclotted blood (Figure 2). The left lobe of the liver was removed by laparoscopy, surgical specimen, and pathologically related photographs (Figure 3). The patient was cured and discharged 10 days after the operation.

Gross examination of the mass showed a diameter of 10.0 cm. On the cut surface, the lesion was gray-yellow and tan, with obvious hemorrhage and necrosis. The boundary of the mass was still clear, with the rupture site located on the liver surface. The liver tissue surrounding the mass was gray-yellow, and no nodules formed. Microscopic examination showed that the peripheral focal bile duct is dilated, accompanied by a cystic adenomatoid change of the bile duct with low-grade intraepithelial neoplasia. A negative liver resection margin was achieved. Immunohistochemistry results indicated HMB-45 (−), CD34 (−), CD117 (−), Dog-1 (−), PCK (−), CD68 (+), CD163 (+), SMA (−), P53 (WT), Ki-67 (+, 5%), ALK (−), Melan A (−), Vim (+), S-100 (−), P63 (−), and Desmin (−). The patient was discharged on the 10th day after surgery. Diagnosis revealed hepatic giant cell tumor with no history of giant cell tumor of bone or other soft tissues. After 2 months of follow-up, the patient was in good condition, and no tumor recurrence or metastasis was found. We arranged for positron emission tomography/CT to be included in the patient’s follow-up plan.

## Discussion

GCT-ST is a fibrous histiocytic tumor that is distinct from other soft tissue giant cell tumors and was previously classified as a low-malignant potential giant cell tumor. It is one of the four entities classified as fibrous histiocytic tumors in the latest edition of the World Health Organization Classification of Soft Tissue Tumors (4). GCT-ST is predominantly benign. The possibility of recurrence and metastasis is extremely low, with a local recurrence rate of 10%–15% (5). Metastasis only occurs in extremely rare cases, mainly in the lungs (6). As tumor-related death appears to be extremely rare, no clinicopathological variables that potentially predict metastasis have been discovered (7).

According to the results of the search literature, our case was the ninth primary giant cell tumor of soft tissues (GCT-ST). Among the nine cases, the age ranged from 46 to 88 years, with a mean of 67.1 years, the ratio of men to women was 1:2, abdominal pain was the most common presenting symptom, hemorrhagic ascites were found in some cases, and the vast majority of patients had no history of malignancy. Most patients had metastatic spread, and the prognosis was poor even after surgical resection, with survival ranging from 32 days to 3 months (8).

In 1972, Guccion and Enzinger published a report that further described the hallmark histological findings of the tumor in addition to its clinical and pathological features (9). Histologically, these lesions show uniformly scattered osteoclast-like giant cells intimately admixed with elliptic to polygonal mononuclear cells and spindle-shaped cells (10); the mononuclear cells have round to oval vesicular nuclei. Osteoclast-like giant cells are evenly distributed throughout the tumor, and their nuclei are similar to those of monocytes (11), showing moderate nuclear atypia and rare mitotic activity (12). These cells are immersed in a connective stroma rich in blood vessels. Tumors are usually well-circumscribed and have abundant blood vessels in the stroma, associated with common hemorrhage, hemosiderin deposition, and accumulation of foam cells (13). Malignant GCT-ST is very rare, characterized by nuclear atypia, pleomorphism, and high mitotic activity of monocytes (5, 6). The term “malignant giant cell tumor of soft parts” should be restricted to histologically high-grade lesions. Immunohistochemically, the osteoclast-like giant cells showed positive immunoreactivity for vimentin and CD68, which are mainly located in osteoclast-like giant cells, highly expressed in mononuclear stromal cells (5). The immunohistochemistry results, on the other hand, for smooth muscle actin, cytokeratin, ALK, CD34, and S-100 are negative. The level of the proliferation index, Ki-67 in our case, was 5%, indicating weak proliferation activity and low-malignant potential.

GCT-ST resembles the giant cell tumor of bone (GCT of bone, GCT-B). GCT-B is an intra- or extra-articular tumor that usually involves tendon sheaths, joints, and bursae, which may erode bone. The molecular pathogenesis of GCT-ST remains obscure. Although GCT-STs have traditionally been considered the soft tissue counterpart of GCT-B, based on the morphological similarities between the two entities, they lack the *H3F3* mutations that characterize their osseous counterparts (13). It has been reported that >90% of GCT-Bs have a driver mutation in the *H3F3A* gene (14), indicating different molecular pathogenesis for the two entities.

Differential diagnoses of GCT-ST include soft tissue mesenchymal tumors that are rich in giant cells, including tenosynovial giant cell tumors and malignant giant cell tumors of soft tissue (15). At the same time, its striking histological similarity to GCT-B can confuse. In addition to significant differences in location, the tenosynovial giant cell tumor (16) also contains a variety of cell groups, including xanthoma cells, siderophages, and lymphocytes. Giant cell malignant fibrous histiocytoma is defined as monocytes and giant cells showing severe atypia, atypical mitosis, and necrosis. Differential diagnoses of malignant entities include giant cell malignant fibrous histiocytoma, leiomyosarcoma rich in osteoclast-like giant cells, epithelioid sarcoma with giant cells, atypical fibroxanthoma with osteoclast-like giant cells, plexiform fibrohistiocytic tumors, and extraskeletal osteosarcoma.

On CT or magnetic resonance imaging, a solid, uneven, often hemorrhagic mass is most common. However, the source of the tumor is not well determined, especially an extended mass located in the left lobe of the liver. Contrast-enhanced ultrasound examination of the liver or endoscopy of the stomach can be further performed. In this case, it was difficult to locate the tumor before surgery. Enhanced abdominal CT indicated that the tumor was located between the liver and stomach, which was highly likely to be derived from the stomach. Combined with that, the gastroscopy revealed gastric submucosal eminence; therefore, preoperative diagnosis was considered a gastric stromal tumor. Due to complicated abdominal bleeding, surgery was performed, discovering that the tumor was from the liver, and the tumor was diagnosed as a hepatic giant cell tumor. On the basis of diagnosis, the following could be concluded: (1) There was no history of giant cell tumor of bone or other soft tissue. Imaging studies showed that the soft tissue mass was not related to bone, synovium, tendon, or joint. (2) The tumor was located in the liver. (3) The histological structure was similar to that of the giant cell tumor of bone. (4) Immunohistochemical staining showed negative epithelial markers of the tumor, excluding hepatocellular carcinoma and stromal tumor, with negative PEComa-related markers.

## Conclusions

GCT-ST is biologically benign with rare local recurrence and distant metastasis (13). Appropriately extended surgical resection is the best treatment. Patients with negative resection margins generally do not relapse. If the tumor can be completely removed and sufficient resection margin can be ensured during surgery, it is expected to be cured (17). GCT-ST should be considered in the differential diagnosis of spindle cell lesions in soft tissue rich in giant cells. Of course, we should make more efforts on how to identify tumors early and enhance screening for diseases, such as regular liver ultrasound or gastroscopy.

## Data Availability

The raw data supporting the conclusions of this article will be made available by the authors, without undue reservation.
